# Account of Diseases in an Eastern District of London, from December 20, 1804, to January 20, 1805

**Published:** 1805-02-01

**Authors:** 


### *Account of Diseases in an Eastern District of London, from December 20, 1804, to January 20, 1805.*

| | |
|---|---|
| ACUTE DISEASES. | |
| Pneumonia | 3 |
| Peripneumonia Notha | 7 |
| Rheumatismus Acutus | 4 |
| CHRONIC DISEASES. | |
| Tussis | 19 |
| Tussis cum Dyspnœa | 33 |
| Dyspnœa | 15 |
| Pleurodyne | 4 |
| Hæmoptysis | 3 |
| Phthisis Pulmonalis | 4 |
| Vermes | 2 |
| Hydrothorax | 3 |
| Ascites | 1 |
| Dyspepsia | 6 |
| Hypochondriasis | 3 |
| Menorrhagia | 5 |
| Chlorosis | 5 |
| Amenorrhœa | 6 |
| Rheumatismus Chronicus | 19 |
| PUERPERAL DISEASES. | |
| Ephemera | 6 |
| Enteritis | 2 |
| Menorrhagia Lochialis | 7 |
| INFANTILE DISEASES. | |
| Convulsio | 3 |
| Pertussis | 1 |
| Aphthæ | 4 |
| Diarrhœa | 6 |

Upon a review of the state of diseases during the past year, we may congratulate the public on having enjoyed a considerable share of health. Since the epidemic of 1803, which extended not only to different parts of this kingdom, but also to neighbouring states, there has been but a comparatively small number of diseases in the metropolis, and their symptoms have been generally mild. Of acute diseases there have been very few. Of typhus, the instances have been comparatively rare. Cholera and dysentery appeared in the autumn; but, in general, in such a form as to occasion very little anxiety to the practitioner; and, as might be expected, the termination was usually favourable.

During the severe cold, which prevailed a few weeks ago, the different complaints of the chest suffered considerable aggravation. Coughs and peripneumonies frequently occurred; their symptoms were severe; and to persons in advanced life the disease generally proved fatal.

